# SLIRP Regulates the Rate of Mitochondrial Protein Synthesis and Protects LRPPRC from Degradation

**DOI:** 10.1371/journal.pgen.1005423

**Published:** 2015-08-06

**Authors:** Marie Lagouge, Arnaud Mourier, Hyun Ju Lee, Henrik Spåhr, Timothy Wai, Christian Kukat, Eduardo Silva Ramos, Elisa Motori, Jakob D. Busch, Stefan Siira, German Mouse Clinic Consortium, Elisabeth Kremmer, Aleksandra Filipovska, Nils-Göran Larsson

**Affiliations:** 1 Department of Mitochondrial Biology, Max Planck Institute for Biology of Ageing, Cologne, Germany; 2 CECAD Research Center, University of Cologne, Cologne, Germany; 3 FACS and Imaging facility, Max Planck Institute for Biology of Ageing, Cologne, Germany; 4 Harry Perkins Institute of Medical Research, Centre for Medical Research and School of Chemistry and Biochemistry, The University of Western Australia, Perth, Australia; 5 German Mouse Clinic, Helmholtz Zentrum München, German Research Center for Environmental Health GmbH, Neuherberg, Germany; 6 Helmholtz Zentrum München, Institute of Molecular Immunology, Munich, Germany; 7 Department of Laboratory Medicine, Karolinska Institute, Stockholm, Sweden; Stanford University School of Medicine, UNITED STATES

## Abstract

We have studied the *in vivo* role of SLIRP in regulation of mitochondrial DNA (mtDNA) gene expression and show here that it stabilizes its interacting partner protein LRPPRC by protecting it from degradation. Although SLIRP is completely dependent on LRPPRC for its stability, reduced levels of LRPPRC persist in the absence of SLIRP *in vivo*. Surprisingly, *Slirp* knockout mice are apparently healthy and only display a minor weight loss, despite a 50–70% reduction in the steady-state levels of mtDNA-encoded mRNAs. In contrast to LRPPRC, SLIRP is dispensable for polyadenylation of mtDNA-encoded mRNAs. Instead, deep RNA sequencing (RNAseq) of mitochondrial ribosomal fractions and additional molecular analyses show that SLIRP is required for proper association of mRNAs to the mitochondrial ribosome and efficient translation. Our findings thus establish distinct functions for SLIRP and LRPPRC within the LRPPRC-SLIRP complex, with a novel role for SLIRP in mitochondrial translation. Very surprisingly, our results also demonstrate that mammalian mitochondria have a great excess of transcripts under basal physiological conditions *in vivo*.

## Author Summary

Mitochondria provide most of the energy required for key metabolic and cellular processes that are essential for life. The biogenesis of the mitochondrial oxidative phosphorylation system, the site of energy conversion, is dependent on the coordinated expression of the mitochondrial and nuclear genomes. Mitochondrial gene expression is largely regulated at the post-transcriptional level by RNA-binding proteins, including the LRPPRC-SLIRP complex. It is still unclear how the proteins within this complex regulate mitochondrial RNA metabolism. Here, we have knocked out the *Slirp* gene in mice to dissect the individual roles of LRPPRC and SLIRP and provide further insights into the mechanisms governing post-transcriptional regulation of mitochondrial gene expression. LRPPRC is required for the maintenance of mitochondrial mRNA polyadenylation whereas SLIRP, by facilitating the presentation (or association) of mRNAs to the mitochondrial ribosome, regulates the rate of translation. In addition, we demonstrate that mitochondrial mRNAs in mammals are present in quantities that far exceed those needed to maintain normal physiology under basal conditions.

## Introduction

Mitochondria are double-membrane bound organelles that have fundamental roles in energy metabolism, cell health and death, making them essential for life. The oxidative phosphorylation (OXPHOS) system is the major site of ATP production in mitochondria and is composed of proteins encoded by two genomes, the nuclear genome and mitochondrial DNA (mtDNA). Consequently coordinated regulation of nuclear and mitochondrial gene expression is required to meet the changing energy demands of the cell. The compact size and organization of mtDNA in animals has necessitated the evolution of unique mechanisms to regulate the expression of the 13 subunits of the OXPHOS system that are mitochondrially encoded. Mitochondrial gene expression is complex and predominantly regulated at the post-transcriptional level [1,2] by nuclear-encoded mitochondrial RNA-binding proteins that control the processing, maturation, translation, stabilization and degradation of mitochondrial RNAs [3]. The mitochondrial RNA polymerase (POLRMT) stimulated by mitochondrial transcription factor A (TFAM) and B2 (TFB2M) produces near-genome length polycistronic transcripts [3]. Because animal mtDNA lacks introns, the 22 mitochondrial tRNA genes that are arranged between the 2 rRNA and 11 mRNA coding genes act as punctuation marks to signal the processing of the polycistronic transcripts [4] by mitochondrial tRNA (mt-tRNA) processing enzymes [5–7]. The processed transcripts undergo extensive maturation, including polyadenylation at the 3′ end of the mitochondrial mRNAs (mt-mRNAs) [8,9], and mitochondrial rRNAs (mt-rRNAs) and mt-tRNAs are modified enzymatically at specific nucleosides to enable proper folding and biogenesis of the translation machinery [2,3]. The matured mt-mRNAs are translated on mitochondrial ribosomes (mitoribosomes) [10], although it is not clear how they are recognized as they lack conventional 5′ and 3′ untranslated regions (UTRs), Shine-Dalgarno sequences and 5′ 7- methylguanosine caps [11].

The mammalian family of RNA-binding pentatricopeptide repeat domain (PPR) proteins consists of seven nuclear-encoded mitochondrial proteins, each of which has a specific role in regulating mitochondrial gene expression from transcription and processing to maturation and translation [12]. The PPR protein LRPPRC first came to attention when a mutation of the *LRPPRC* gene was shown to cause a rare French-Canadian variant of Leigh syndrome characterized by cytochrome c oxidase deficiency [13]. In cultured cells, *LRPPRC* knockdown (KD) causes a reduction of mt-mRNA levels [14–16] and impaired mitochondrial translation [16]. LRPPRC physically interacts with SLIRP, which has an RNA recognition motif (RRM), consistent with a role in mitochondrial RNA metabolism [14]. LRPPRC and SLIRP form a complex that mediates mt-mRNA stability [15–17] and both proteins are co-stabilized within this complex because reduction of LRPPRC levels leads to concomitant reduction of SLIRP [14–18]. In mice, the LRPPRC-SLIRP complex regulates mt-mRNA stability, polyadenylation and coordinated mitochondrial translation [17]. We have also demonstrated that the bicoid stability factor (BSF, renamed DmLRPPRC1), one of the two *Drosophila melanogaster* orthologues of mammalian LRPPRC [19,20], has a very similar function as the mammalian one [21]. Furthermore, DmLRPPRC1 associates with one of the two fly orthologues of SLIRP [19,21], suggesting that the interaction between PPR-motif- and RRM-containing proteins is important for mitochondrial RNA metabolism and has been conserved through evolution.

To address the unclear *in vivo* role of SLIRP and its function within the LRPPRC-SLIRP complex, we generated *Slirp* knockout mice. Molecular analyses revealed that SLIRP is required to stabilize LRPPRC. In addition, our findings show that LRPPRC and SLIRP have distinct roles within the mt-mRNA-stabilizing complex they form, *i*.*e*. LRPPRC is required for maintenance of polyadenylation whereas SLIRP regulates the rate of translation. Very surprisingly, we also report that mice lacking SLIRP are apparently healthy despite a very drastic (50–70%) depletion of mt-mRNAs. These findings show that mt-mRNAs in mammalian mitochondria are present in quantities that far exceed those needed to maintain normal physiology.

## Results

### Generation of *Slirp* knockout mice

In mammals SLIRP forms a complex with the mitochondrial protein LRPPRC [16–18] and the complex is required for the stability of mt-mRNAs, polyadenylation and coordinated mitochondrial translation [15–17]. SLIRP is predicted to localize to mitochondria with a probability of 94.4% using the MitoProtII software [22] and we confirmed this prediction by using immunocytochemistry to show that endogenous SLIRP co-localizes with the mitochondrial ATPase complex (S1A Fig) in 143B cells.

To investigate the specific role of SLIRP within mitochondria *in vivo*, we generated a germline *Slirp* knockout (KO) mouse model (*Slirp* ^-/-^) *via* excision of the floxed exon 2 of *Slirp* by expressing the Cre-recombinase under the control of the β-actin promoter (S1B Fig). The resulting *Slirp* ^+/-^ mice (S1C Fig) were inter-crossed to generate *Slirp* ^-/-^ mice and all expected genotypes were obtained at Mendelian ratios, thus showing that SLIRP, in contrast to LRPPRC [17], is not required for embryonic development. Mice lacking SLIRP were apparently healthy with no obvious phenotype, except a slight reduction in body weight (S1D Fig). In contrast to a previous report [23], we also found that lack of SLIRP does not impair fertility as crosses between *Slirp* ^-/-^ males or females and wild-type mice produced normal litter sizes. These findings show that the *in vivo* roles of SLIRP and LRPPRC are at least partly divergent.

### SLIRP is necessary for the stability of mitochondrial mRNAs *in vivo*

**Fig 1:**
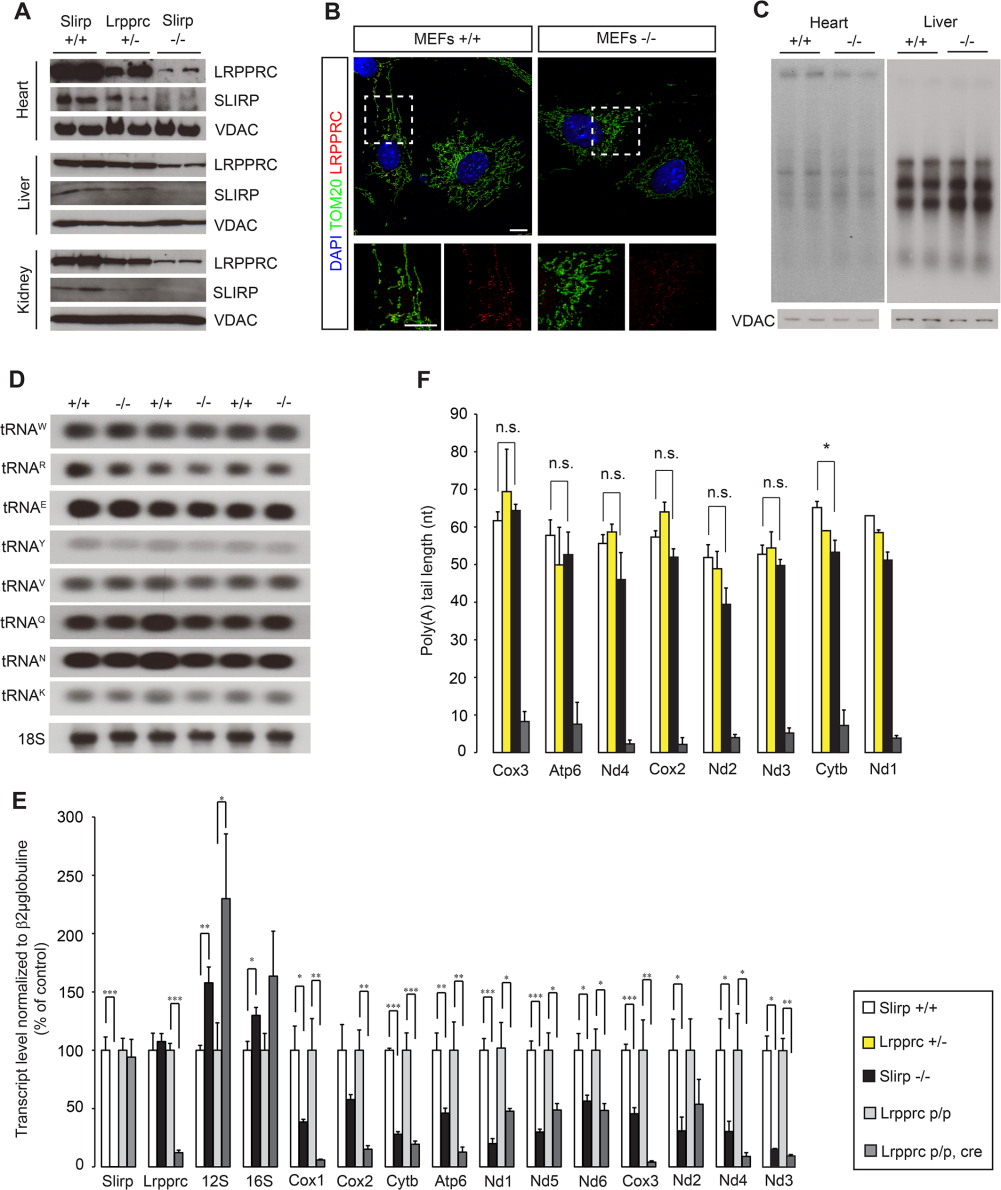
Loss of SLIRP compromises the stability of mitochondrial mRNAs and LRPPRC. (A) Immunoblot of SLIRP and LRPPRC protein levels in heart, liver and kidney mitochondria from 12-week old wild-type (*Slirp* ^+/+^), *Slirp* homozygous knockout (KO, *Slirp* ^-/-^) and *Lrpprc* heterozygous KO (*Lrpprc* ^+/-^) mice. VDAC was used as a loading control. (B) Representative confocal microscopy images of *Slirp* ^+/+^ (left) and *Slirp* ^-/-^ (right) MEFs, stained for LRPPRC and TOM20 as a mitochondrial marker. Magnifications of the dashed boxed areas show merged channels of LRPPRC and TOM20 (bottom, left) and single channels of LRPPRC (bottom, right). Scale bars presented, 10 μm. (C) *In organello de novo* transcription assays performed on heart and liver mitochondria isolated from 12-week old *Slirp* ^+/+^ and *Slirp* ^-/-^ mice. VDAC was used as a loading control. (D) Mitochondrial tRNA steady-state levels assessed by northern blotting in hearts of 12-week old *Slirp* ^+/+^ *and Slirp* ^-/-^ mice. (E) Mitochondrial transcript steady-state levels assessed by qRT-PCR in hearts from 12-week old *Slirp* ^+/+^ (white bars) and *Slirp* ^-/-^ (black bars) mice, as well as in hearts from 12-week old *Lrpprc* control (*Lrpprc* p/p, light grey bars) and conditional KO (*Lrpprc* p/p, cre, dark grey bars), n = 5. Error bars represent SEM. * p value < 0.05. ** p value < 0.01. *** p value < 0.001. (F) Measurement of mitochondrial transcript poly(A) tail length from heart mitochondria of *Slirp* ^+/+^ (white bars), *Slirp* ^-/-^ (black bars), *Lrpprc* ^+/-^ (yellow bars) and *Lrpprc* p/p, cre (grey bars) mice. Error bars represent SEM. n.s. means not significant. * p value < 0.05.

Steady-state SLIRP levels have been shown to correlate with those of LRPPRC [14–16] and conditional KO of *Lrpprc* causes complete loss of SLIRP [17]. Therefore, we investigated LRPPRC levels by immunoblotting of mitochondria isolated from *Slirp* ^-/-^ mice and found that ~25% of the LRPPRC protein remained in heart, liver and kidney of these mice (Fig 1A). The reduction of LRPPRC protein levels upon *Slirp* deletion was also confirmed by immunocytochemistry on mouse embryonic fibroblasts (MEFs) derived from *Slirp* ^-/-^ mice (Fig 1B). Our findings thus show that low levels of LRPPRC can be maintained without forming a complex with SLIRP, whereas in wild-type mice LRPPRC and SLIRP form a stable complex in both heart and liver, as confirmed by co-immunoprecipitation (S1E Fig). We found that LRPPRC is reduced by ~50% in heart, liver and kidney mitochondria in *Lrpprc* ^+/-^ mice, consistent with our previous results [24], and these levels were further reduced to ~25% in the *Slirp* ^-/-^ mice (Fig 1A). This finding shows that low levels of LRPPRC are sufficient for healthy survival in mice lacking SLIRP, and that SLIRP, which requires LRPPRC for its stability, may have acquired a role in fine-tuning mitochondrial gene expression.

We proceeded to investigate how loss of SLIRP will impact mitochondrial gene expression and found normal levels of mtDNA in *Slirp* ^-/-^ mitochondria (S2A Fig). Also the transcription rates, measured by *de novo* transcription assays, were normal in liver mitochondria and slightly decreased in heart mitochondria, in the absence of SLIRP (Fig 1C). The occurrence of normal *de novo* transcription was further confirmed by the finding of unaltered steady-state levels of mature mt-tRNAs in *Slirp* ^-/-^ hearts (Fig 1D). In contrast, the steady-state levels of mt-mRNAs were strikingly reduced in heart (Figs 1E and S2B) and liver (S2C Fig) mitochondria isolated from *Slirp* ^-/-^ mice, consistent with *in vitro* results from *SLIRP* KD in cultured cells [14–16]. Our finding of reduced mt-mRNA levels despite normal *de novo* transcription shows that the mt-mRNA stability is reduced in the absence of SLIRP.

Next, we compared the steady-state levels of mt-mRNAs in hearts of *Slirp* ^-/-^ and *Lrpprc* conditional KO mice at 12 weeks (Figs 1E and S2B), which is the age at which the *Lrpprc* conditional KO mice start to die [17]. We found less pronounced decrease of mt-mRNA steady-state levels, with the exception of Nd1, Nd2 and Nd5, in the *Slirp* ^-/-^ hearts in comparison with *Lrpprc* conditional KO hearts (Figs 1E and S2B). This differential decrease in mt-mRNA stability could be accounted for by the fact that there is a remaining fraction of LRPPRC protein in the *Slirp* ^-/-^ mice, whereas LRPPRC is completely absent in the hearts of *Lrpprc* conditional KO mice. It is surprising that the *Slirp* ^-/-^ mice are apparently healthy, with the exception of a slight weight loss (S1D Fig), despite such profound reduction (50–70%) of the mt-mRNA steady-state levels in all investigated tissues. This finding shows that mt-mRNAs are in significant excess *in vivo* and suggests that respiratory chain (RC) dysfunction only occurs if the transcript levels drop below a certain minimal threshold.

### SLIRP does not affect the polyadenylation status of mitochondrial mRNAs

In mammalian mitochondria, mRNAs, with the exception of Nd6, contain short poly(A) tails [8,9], which are necessary to complete the termination codon for seven of the total 11 mt-mRNAs. The LRPPRC-SLIRP complex, which is involved in mt-mRNA stability, has been found to maintain polyadenylation [15,17], but the role for poly(A) tails in the regulation of mt-mRNA stability is unclear. However, polyadenylation appears to have roles in mitochondrial translation that are distinct from termination codon formation [25,26], which is consistent with the requirement of LRPPRC for coordinated translation in mammalian mitochondria [16,17]. Interestingly, we found that poly(A) tail length was intact in the absence of SLIRP (Figs 1F and S2D), which shows that SLIRP is not required for the *in vivo* maintenance of mitochondrial polyadenylation. This finding also demonstrates that the presence of poly(A) tails is not sufficient to ensure mt-mRNA stability *in vivo* when SLIRP is lost (Fig 1E and 1F). LRPPRC has been shown to promote polyadenylation by the mitochondrial poly(A) polymerase [15,27] and our findings show that normal poly(A) tail length can be maintained even if the levels of LRPPRC are low, as it is the case in the *Lrpprc* ^+/-^ and *Slirp* ^-/-^ mice (Fig 1A and 1F). A corollary of this is that SLIRP may have an additional function besides maintaining mt-mRNA stability as part of the LRPPRC-SLIRP complex.

### Loss of SLIRP affects the engagement of mitochondrial mRNAs with the mitochondrial ribosome

Next we investigated how decreased levels of mt-mRNAs affected the protein synthesis machinery in mitochondria from *Slirp* ^-/-^ mice. We measured the steady-state levels of the mitochondrial 12S and 16S rRNAs and found that they were increased in *Slirp* ^-/-^ relative to control mice (Figs 1E and S2B and S2C). The increase in 16S rRNA correlated with an increased amount of MRPL37, a mitochondrial ribosomal protein (MRP) of the large subunit, in *Slirp* ^-/-^ heart and liver mitochondria (S3A Fig). This apparent increase in mitoribosome biogenesis is presumably a compensatory response to the reduced mt-mRNA stability observed upon SLIRP loss.

**Fig 2:**
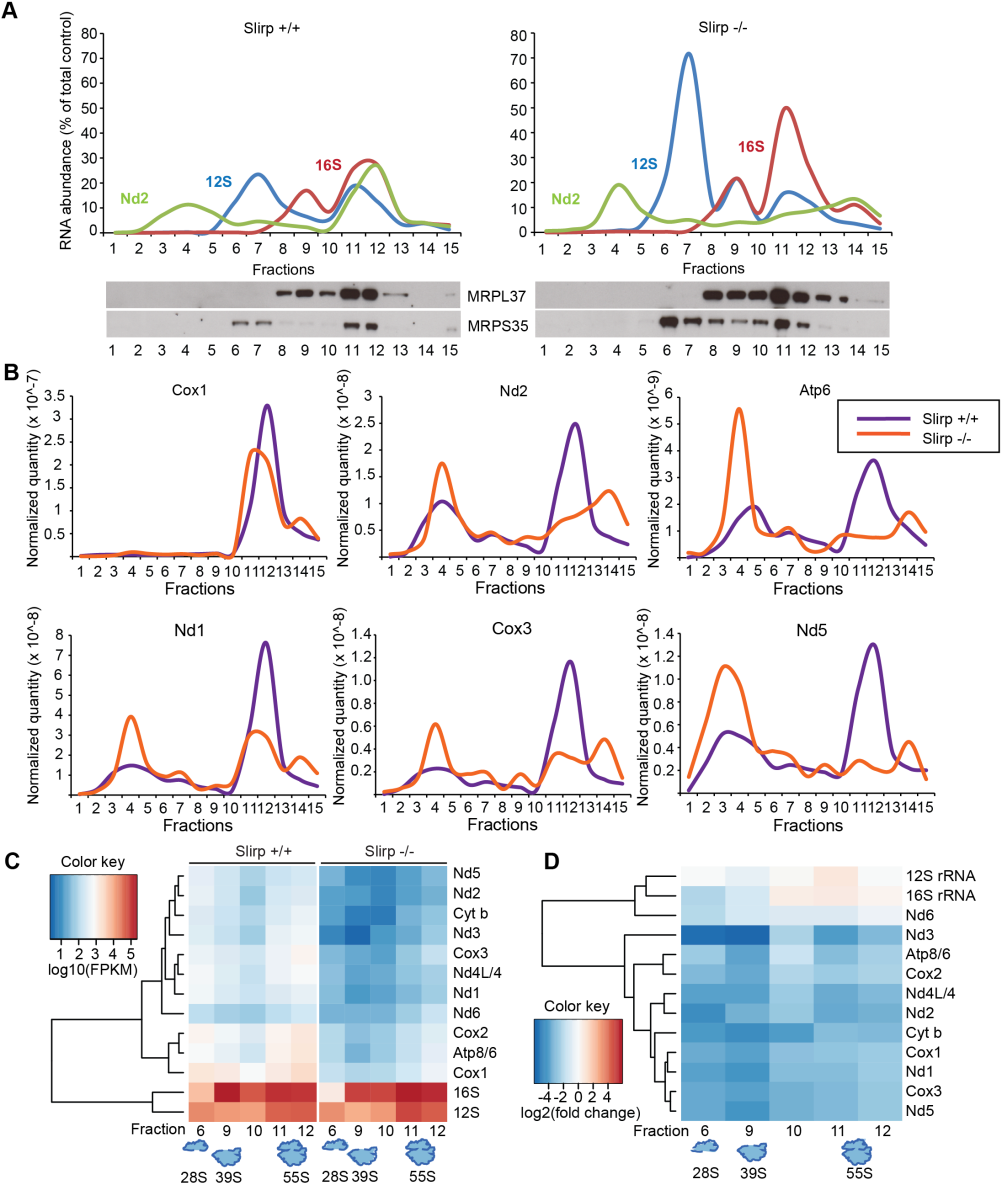
SLIRP loss affects the engagement of mitochondrial mRNAs with the mitochondrial ribosome. (A) Sedimentation profile in sucrose density gradient of transcripts and ribosomal proteins from liver mitochondria from 12-week old wild-type (*Slirp* ^+/+^, left panel) and *Slirp* homozygous knockout (*Slirp* ^-/-^, right panel) animals. Individual mitochondrial transcripts were detected by qRT-PCR. The abundance of a given RNA in each fraction is shown as percentage of the total level in the control. The migration of the small (28S) and large (39S) mitochondrial ribosomal subunits, and of the assembled mitochondrial ribosome (55S) is assessed by the profiles of the 12S (blue line) and 16S (red line) rRNAs as well as by the migration of subunit-specific ribosomal proteins (MRPL37 and MRPS35) detected by immunoblotting. (B) Individual mRNA sedimentation profiles from the gradient described in (A). *Slirp* ^+/+^ profiles are depicted in purple and *Slirp* ^-/-^ profiles are depicted in orange. Individual mitochondrial mRNAs were detected by qRT-PCR and the mRNA distribution profile is shown after normalization to controls, *i*.*e*. the quantity of a given mt-mRNA, named RNAx, in each fraction of the *Slirp* ^-/-^ gradient was normalized to the total RNAx_Slirp+/+_/total RNAx_Slirp-/-_ ratio, where total RNAx is the sum of the RNAx quantity detected across all the fractions. (C) Hierarchical clustered expression levels of mitochondrial transcripts (log10FPKM) across all fractions from both *Slirp* ^+/+^ and *Slirp* ^-/-^ mitochondria. (D) Hierarchical clustered log2 fold changes in transcript expression for each fraction, showing the overall decrease in mRNA levels of *Slirp* ^-/-^ compared to *Slirp* ^+/+^ mitochondria.

To assess the state of association of mt-mRNAs with the mitoribosome we performed sucrose sedimentation gradient analyses of mitochondrial extracts. We used qRT-PCR to determine the sedimentation profile of the small (28S) and large (39S) ribosomal subunits and the fully assembled mitoribosome (55S) (Figs 2A and S3C). The 12S rRNA co-migrated with the MRPS35 protein of the 28S subunit and the 16S rRNA co-migrated with MRPL37 (Figs 2A and S3C, left panels), which shows that the 28S subunit was mainly present in fractions 6–7, the 39S subunit in fraction 9 and the 55S mitoribosome in fractions 11–12 in control mice. Strikingly, in *Slirp* ^-/-^ mitochondria of liver (Fig 2A, right panel) and heart (S3C Fig, right panel), the ribosome profiles were altered as shown by the continuous distribution of MRPL37 and 16S rRNA between fractions 9 and 12. This continuous distribution may occur as a consequence of the increased steady-state levels of mt-rRNAs and MRPs (S3A Fig). We also measured the abundance of mt-mRNAs in the different fractions of the gradient by qRT-PCR and could identify two distinct pools, one translationally inactive in fractions 4–5 and a second one, translationally active, that co-migrates with the assembled mitoribosome (Figs 2A and S3C). To investigate the proportion of mt-mRNAs engaged with the mitoribosome without being misled by the global decrease of mt-mRNA levels in *Slirp* ^-/-^ mice, we normalized *Slirp* ^-/-^ mt-mRNA levels to those of the control samples (Fig 2B). Interestingly, after normalization, we found that mt-mRNAs were less engaged with the assembled mitoribosome in the *Slirp* ^-/-^ liver mitochondria in comparison with controls (Fig 2B). Strikingly, the profile was the opposite in heart where we found increased engagement of mt-mRNAs with the 55S mitoribosome in the *Slirp* ^-/-^ heart mitochondria in comparison with controls (S3D Fig).

Next we investigated the association of mt-mRNAs with the mitoribosome by performing RNA sequencing (RNAseq) of fractions from liver mitochondria that corresponded to the 28S and 39S subunits and to the 55S mitoribosome. In addition we carried out RNAseq of the fractions between the 39S subunit and the 55S mitoribosome, as we observed a continuous distribution of large subunit proteins and rRNA in this region of the gradient in *Slirp* ^-/-^ mitochondria. Differential expression analyses of the mt-mRNAs indicate a global and dramatic decrease of their abundance across the ribosomal fractions in liver mitochondria where SLIRP is lost (Fig 2C), which is in line with the reduced mt-mRNA steady-state levels previously assessed (S2C Fig). The levels of the Nd6 mt-mRNA associated with the mitoribosome were not affected by the loss of SLIRP (Fig 2C), suggesting that the association of Nd6 with the mitoribosome is possibly independent from the LRPPRC-SLIRP complex. Furthermore, both *Slirp* ^+/+^ and *Slirp* ^-/-^ datasets revealed that mt-mRNAs preferentially co-migrate with the 55S mitoribosome and with the 28S subunit as most mt-mRNAs were found in fractions 6 and 11–12 (Fig 2C). We observed a greater enrichment of mt-mRNAs, albeit to varying extents for different mt-mRNAs, with the 28S subunit compared to the 39S subunit, indicating that mt-mRNAs engage the small subunit initially, as is the case for bacterial and cytoplasmic ribosomes [28]. This trend was also observed in the *Slirp* ^-/-^ datasets, despite the significant reduction in mt-mRNAs (Fig 2C). The levels of mitochondrial transcripts in *Slirp* ^-/-^ ribosomal fractions relative to the levels in the corresponding *Slirp* ^+/+^ fractions (Fig 2D), confirmed the global decrease in the abundance of all mt-mRNAs across those fractions, with the exception of Nd6. In addition we confirmed the increase in the mt-rRNA levels in *Slirp* ^-/-^ liver mitochondria, as previously shown by qRT-PCR (S2C Fig). Furthermore we observed a reduced presence of mt-mRNAs in fractions 11 and 12 (Fig 2D), confirming our finding that mt-mRNAs were less engaged with the assembled mitoribosome in the *Slirp* ^-/-^ liver mitochondria in comparison with controls (Fig 2B). Interestingly, the greatest decrease in mt-mRNA levels was found in fractions 6 and 9 (Fig 2D), suggesting that loss of SLIRP may affect the ordered association or disassembly of the ribosomal components with mt-mRNAs and that SLIRP may have a role in regulating the presentation of mature mt-mRNA to the mitochondrial ribosome.

### SLIRP fine-tunes the rate of mitochondrial protein synthesis

**Fig 3:**
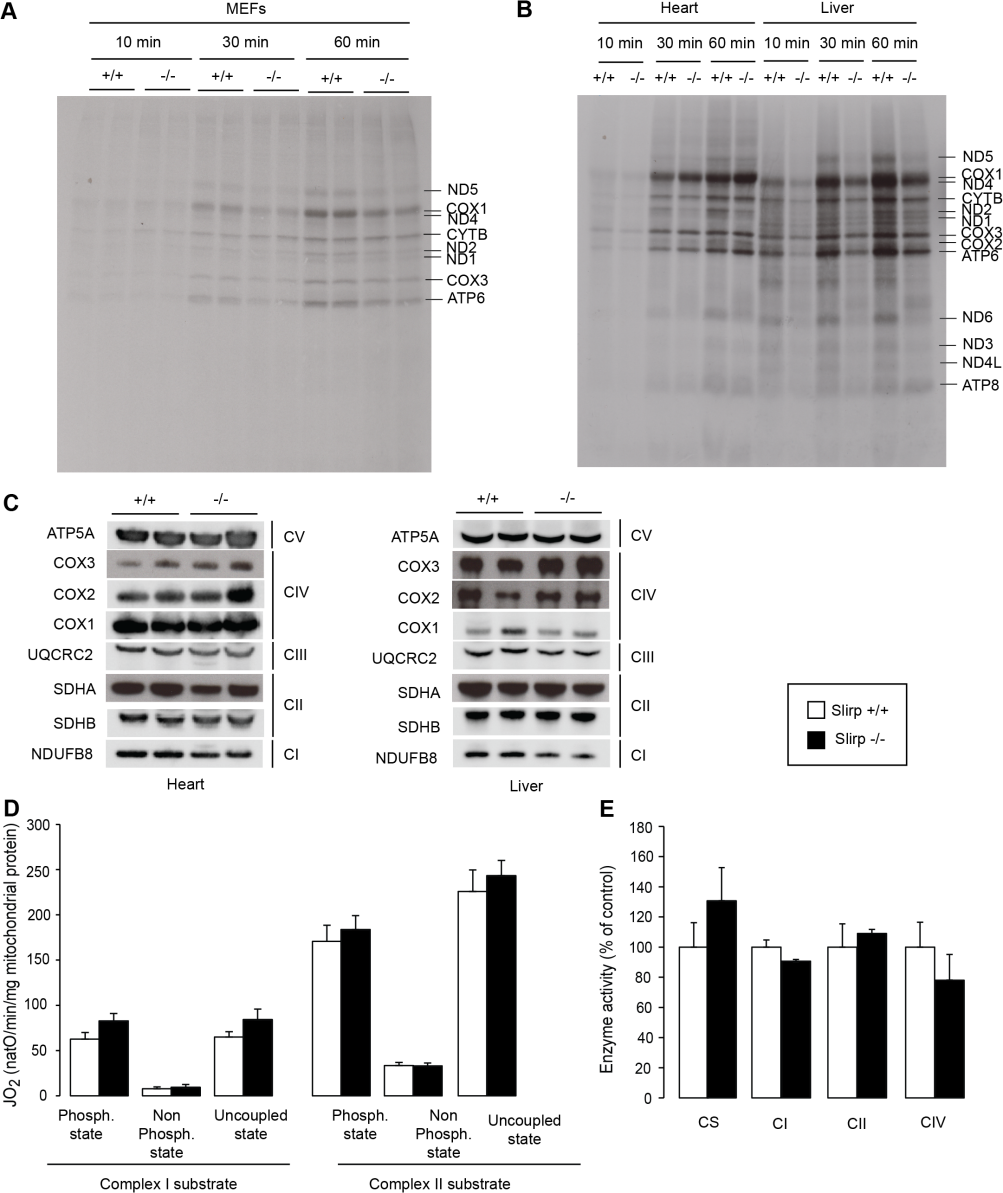
SLIRP regulates the rate of translation but is not essential for respiratory chain activity. (A) Mitochondrial translation rate assessed by *in cellulo* ^35^S-methionine/cysteine pulse labelling for 10, 30 and 60 minutes in wild-type (*Slirp* ^+/+^) and *Slirp* homozygous knockout (*Slirp* ^-/-^) primary MEFs. (B) Mitochondrial translation rate assessed by *in organello* ^35^S-methionine pulse labelling for 10, 30 and 60 minutes in isolated heart and liver mitochondria from 12-week old *Slirp* ^+/+^ and *Slirp* ^-/-^ mice. (C) Steady-state levels of the mitochondria- and nucleus-encoded subunits of the respiratory chain complexes as assessed by western blot analysis of protein extracts from heart and liver mitochondria from 12-week old *Slirp* ^+/+^ and *Slirp* ^-/-^ mice. (D) Oxygen consumption rate of isolated liver mitochondria from 12-week old *Slirp* ^+/+^ (white bars) and *Slirp* ^-/-^ (black bars) mice. Isolated mitochondria were incubated with complex I or complex II substrates. Each set of substrates was successively combined with ADP (to assess the phosphorylating respiration), oligomycin (to assess the non-phosphorylating respiration) and CCCP (to assess uncoupled respiration). n = 3. Error bars represent SEM. (E) Activity of the respiratory chain complexes I (CI), II (CII) and IV (CIV) of liver mitochondria from 12-week old *Slirp* ^+/+^ and *Slirp* ^-/-^ mice. Citrate synthase activity (CS) was used as a control. n = 3. Error bars represent SEM.

We proceeded to measure mitochondrial protein synthesis to assess the biological significance of the altered engagement of mt-mRNAs with the mitoribosome in the absence of SLIRP. We determined the rate of translation by following ^35^S-methionine incorporation into newly synthetized mitochondrial polypeptides over time in MEFs (Fig 3A) and in isolated heart, liver (Fig 3B) and kidney (S4A Fig) mitochondria. Interestingly, we found that the translation rate was impaired in the *Slirp* ^-/-^ MEFs as well as liver and kidney mitochondria (Figs 3A and S4A), which is in line with the observed reduced engagement of the mt-mRNAs with the mitoribosome (55S) in *Slirp* ^-/-^ liver mitochondria (Fig 2B and 2C). In contrast, in *Slirp* ^-/-^ heart mitochondria the incorporation of ^35^S-methionine was comparable to that of control heart mitochondria (Fig 3B), with the exception of Nd2 and Cox1/Nd4 whose translation seemed to be affected by the loss of SLIRP. The maintenance of a comparable translation rate despite *Slirp* knockout is consistent with the observed increased engagement of mt-mRNAs with the 55S mitoribosome in *Slirp* ^-/-^ hearts (S3D Fig). These findings suggest that SLIRP is involved in presenting mature mRNAs to the mitoribosome in order to promote mitochondrial translation, but that its loss can be compensated for in certain tissues such as the heart. We found that the steady-state levels of the mitochondrial translation initiation factor 3 (mtIF3) were increased in *Slirp* ^-/-^ mitochondria in comparison with controls, especially in the liver (S3B Fig), which likely constitutes a compensatory response to the impaired rate of translation. The tissue-specific mitochondrial translation defect, which is very minor in the heart and more apparent in the liver and kidney, does not seem to impact the assembly of the RC subunits, as their steady-state levels are similar in liver and heart mitochondria from control and *Slirp* ^-/-^ mice (Fig 3C). Furthermore the respiration was normal under phosphorylating, non-phosphorylating, and uncoupled conditions in *Slirp* ^-/-^ mitochondria from liver (Fig 3D) and heart (S4C Fig). Consistently, the RC enzyme activities of complexes I, II and IV were comparable in mitochondria from liver (Fig 3E) and heart (S4D Fig) in *Slirp* ^-/-^ and control mice.

Together, these data argue that SLIRP can act as a general activator of mitochondrial translation, whose loss (i) can be overcome by an unknown mechanism in tissues such as the heart and (ii) is not sufficient to induce OXPHOS dysfunction in tissues where translation rate is affected such as the liver and kidney. We hypothesize that despite their reduced stability and impaired loading onto the mitoribosome, mt-mRNAs in *Slirp* ^-/-^ mice are still translated at rates sufficient to preserve normal OXPHOS activity, which likely explains the absence of a pathophysiology in the *Slirp* ^-/-^ mice under basal conditions. In addition, the stability of the mitochondria-encoded RC subunits as observed by ^35^S-methionine pulse-chase assay in the *Slirp* ^-/-^ MEFs (S4B Fig) is likely contributing to the maintenance of normal OXHPOS function despite the decrease in translation.

### Stabilization of LRPPRC cannot rescue mitochondrial mRNA stability in the absence of SLIRP

**Fig 4:**
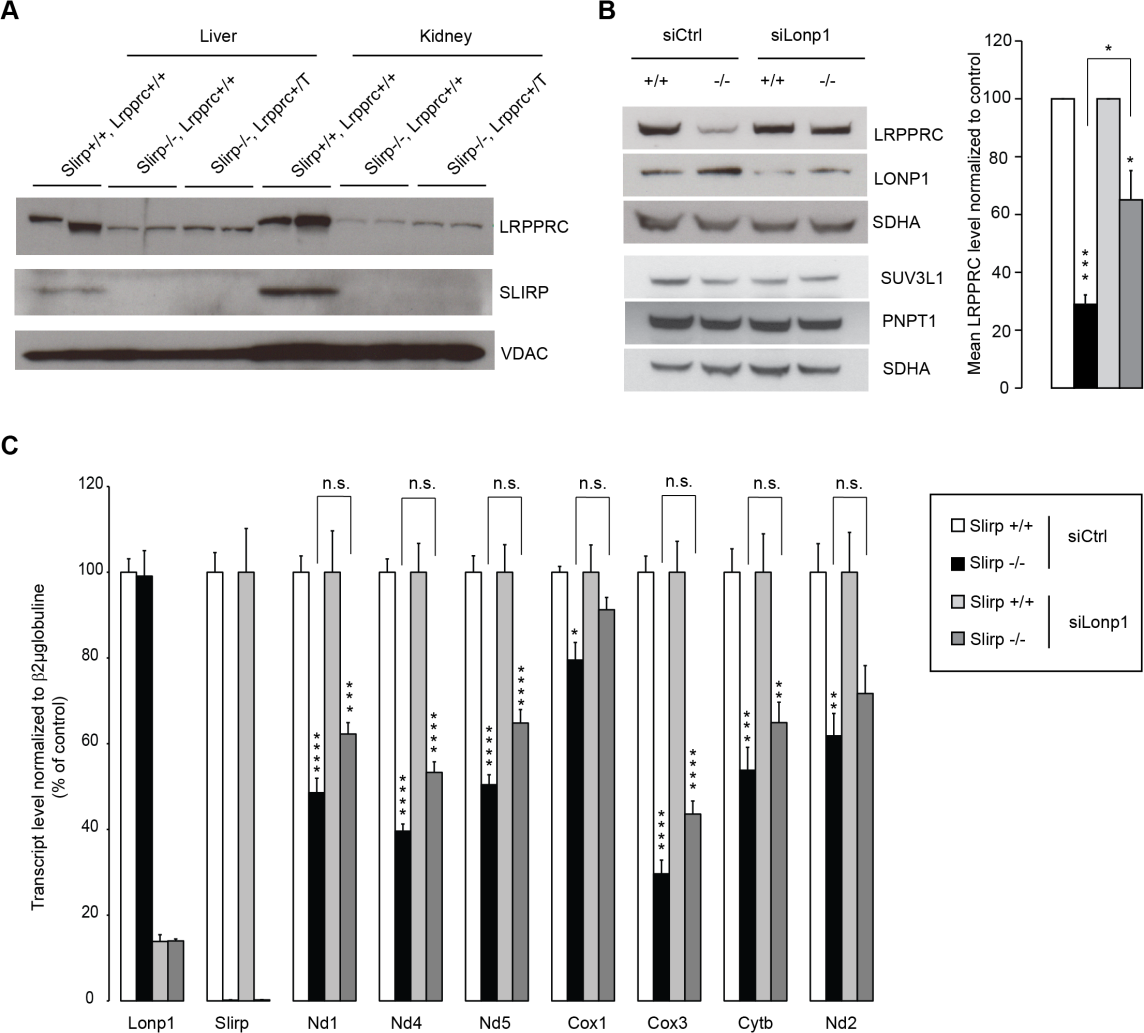
LRPPRC is degraded by LONP1 in the absence of SLIRP and LRPPRC alone cannot preserve mitochondrial transcript stability. (A) Immunoblotting of SLIRP and LRPPRC protein levels in liver and kidney mitochondria from 12-week old wild-type (*Slirp* ^+/+^, *Lrpprc* ^+/+^) and *Slirp* homozygous knockout (*Slirp* ^-/-^, *Lrpprc* ^+/+^) mice and mice overexpressing *Lrpprc* on a *Slirp* homozygous knockout background (Slirp^-/-^, Lrpprc^+/T^). VDAC was used as a loading control. (B) Immunoblotting of LRPPRC protein levels in *Slirp* ^+/+^ and *Slirp* ^-/-^ primary MEFs after transfection with a siRNA directed against the expression of the LONP1 protease (siLonp1) or with a scrambled siRNA (siCtrl). LONP1 was detected to assess the efficiency of the knockdown (KD), SUV3L1 and PNPT1 were detected to assess the steady-state level of the mitochondrial RNA degradosome and SDHA was used as a loading control (left panel). The right panel represents the quantification of three independent experiments. Error bars represent SEM. * p value < 0.05. *** p value < 0.001. (C) QRT-PCR analysis of the mitochondrial transcript steady-state levels after KD of *Lonp1* expression in *Slirp* ^+/+^ and *Slirp* ^-/-^ MEFs as described in (B). n = 6. Error bars represent SEM. * p value < 0.05. ** p value < 0.01. *** p value < 0.001. n.s. means not significant.

In plants, PPR proteins have been found to associate via protein-protein interactions with additional RNA-binding proteins, including RRM proteins to regulate gene expression [29]. The co-stabilization of SLIRP and LRPPRC as a complex [16–18] has provided a challenge to specifically decipher their individual roles in mitochondria. Our data suggest that the decreased levels of mt-mRNA in *Slirp* ^-/-^ mitochondria could possibly be a consequence of the decreased LRPPRC protein levels, where SLIRP could act as a stabilizing partner for LRPPRC without directly affecting mt-mRNA stability. To further investigate this hypothesis, we generated mice ubiquitously overexpressing *Lrpprc* on a *Slirp* KO background (Fig 4A), in an attempt to overcome the co-stability dependence of the two proteins. Interestingly, we found that LRPPRC protein levels could not be restored in the absence of SLIRP thus confirming that SLIRP is essential for LRPPRC protein stabilization. Mitochondrial protein turnover is regulated by several proteases [30], among which LONP1 has been shown to target components of the mitochondrial gene expression machinery [31,32]. By knocking down the expression of *Lonp1* in *Slirp* ^-/-^ MEFs we could partially restore LRPPRC protein levels, demonstrating that LRPPRC is targeted for degradation by this matrix protease in the absence of SLIRP (Fig 4B). We used this rescue model to determine if the increased steady-state levels of LRPPRC would restore mt-mRNA levels in the absence of SLIRP (Fig 4C). However, the significant rescue of LRPPRC steady-state levels induced by the *Lonp1* KD (Fig 4B) did not significantly increase mt-mRNA levels (Fig 4C), yet in the absence of any adverse effect on the mt-RNA degradation machinery (Fig 4B) [33]. In previous work we have shown that *Lrpprc* ^+/-^ mice, that have a ~50% reduction of LRPPRC protein levels, have normal mitochondrial transcript stability [24]. In contrast, we show here that a similar LRPPRC level reduction combined with loss of SLIRP, as seen in *Slirp* ^-/-^ MEFs upon *Lonp1* KD (Fig 4B), induced a significant reduction in the steady-state levels of mitochondrial transcripts (Fig 4C). Taken together, these results show that SLIRP has a role in mt-mRNA stability that can be disconnected from its function in stabilizing LRPPRC. We thus conclude that both LRPPRC and SLIRP are required for maintaining mt-mRNA steady-state levels independent of their roles in stabilizing the other partner of the LRPPRC-SLIRP complex.

## Discussion

It has previously been shown that the stability of SLIRP is absolutely dependent on the presence of LRPPRC [14–18]. Here, we show that a small fraction of LRPPRC can be maintained even if SLIRP is absent. It should be noted that SLIRP is necessary for maintaining normal levels of LRPPRC, which can otherwise be degraded by mitochondrial matrix proteases such as LONP1. Beyond their roles in co-stabilization, SLIRP and LRPPRC share a common direct role on mitochondrial transcript stability as we have shown that both proteins are required to maintain mt-mRNA steady-state levels. Indeed, mt-mRNA stability could not be restored by the sole rescue of LRPPRC levels in the absence of SLIRP. An alternative hypothesis is that the rescued LRPPRC is not fully functional in the absence of SLIRP and can therefore not fulfill its mt-mRNA stabilizing function. This is however very unlikely as we have shown that low levels of LRPPRC, independent of the LRPPRC-SLIRP complex, are sufficient, and therefore functional, for mt-mRNA poly(A) tail maintenance.

Interestingly we show that in contrast to its partner LRPPRC, SLIRP is not involved in the maintenance of the poly(A) tails of mt-mRNAs *in vivo*. This result was surprising given the reduced poly(A) tail abundance and subsequent accumulation of mt-mRNA oligo(A) tails reported upon *SLIRP* KD in cells [15], but was in line with the observation by the same authors that LRPPRC alone could stimulate mt-mRNA polyadenylation *in vitro* and that SLIRP only had a supportive role in this assay through the stabilization of LRPPRC [15]. However this last observation contrasts with an *in vitro* study showing that the extension of the poly(A) tail was enhanced when LRPPRC was complexed with SLIRP, compared to LRPPRC alone [27]. This is likely because recombinant PPR proteins can be unstable and prone to precipitation [34], and LRPPRC would require SLIRP for its *in vitro* stability and thereby would enhance its intrinsic activity required for poly(A) tail maintenance.

We find that SLIRP is not involved in poly(A) tail maintenance *in vivo*, but instead has a role in fine-tuning the rate of mitochondrial protein synthesis. Indeed, we have shown using RNAseq that SLIRP can globally facilitate the ordered association of mature mt-mRNAs with mitoribosome components, thereby affecting the rate of translation. The only exception was Nd6 mRNA, whose stability required the presence of LRPPRC and SLIRP but whose engagement with the mitoribosome seemed in contrast to be unaffected by the loss of SLIRP. Whether this independence is conferred by the absence of a poly(A) tail is not clear. Furthermore, as mentioned above, the residual levels of LRPPRC are sufficient to stabilize the poly(A) tails of mt-mRNAs, enabling normal protein synthesis in the heart but not in the liver and kidney. This effect is independent of the interaction between LRPPRC and SLIRP as we have confirmed that this complex is present both in mouse heart and liver mitochondria. The apparent tissue-specific effect of SLIRP loss on mitochondrial translation could be explained by an unknown mechanism compensating for the absence of SLIRP in the heart. We found that the translation rate was also impaired in the *Slirp* ^-/-^ MEFs and we therefore hypothesize that the consequence of SLIRP loss could be linked to the proliferative status of the tissue. Indeed, faster rates of mitochondrial translation may be required in proliferating cells such as hepatocytes and MEFs. The role of SLIRP in maintaining the translation rate would therefore be better illustrated in proliferative tissues, where its absence would confer a more obvious disadvantage. However, irrespective of the effects on protein synthesis, polypeptides are made in sufficient amounts for proper assembly of the OXPHOS complexes and SLIRP loss does not compromise mitochondrial respiration. The observation that a moderate decrease in mitochondrial translation does not lead to a reduced abundance of the steady state levels of mitochondria-encoded RC subunits is likely due to the stability of the RC subunits.

Notably, mice lacking SLIRP are apparently healthy, with the exception of a slight weight loss, despite having a profound (50–70%) depletion of mt-mRNAs. The levels of the mtDNA-encoded mt-mRNAs are thus present in great excess under normal physiological conditions. It is interesting to speculate that the excess of transcripts would enable robust and rapid activation of mitochondrial protein synthesis in response to sudden changes in metabolic demand. Our findings also indicate that excess mt-mRNAs could provide a buffer that can cope with dramatic reduction of transcription of mtDNA-encoded genes, as might occur when mtDNA undergoes replication in rapidly dividing cells.

## Materials and Methods

### Generation of *Slirp* knockout mice

The targeting vector for disruption of *Slirp* in ES cells (derived from C57BL/6N mice) was generated by using BAC clones from the C57BL/6J RPCI-23 BAC library by Taconic Artemis. To generate a conditional *Slirp* knockout (KO) allele, exon 2 of the *Slirp* locus was flanked by loxP sites. The puromycin resistance marker (PuroR) was flanked by F3 sites and inserted into intron 2. The puromycin resistance cassette was removed by mating *Slirp* ^+/loxP-Puro^ mice with transgenic mice ubiquitously expressing Flp recombinase. The resulting *Slirp* ^+/loxP^ mice were mated with mice ubiquitously expressing the Cre recombinase under the dependence of the β- actin promoter to generate *Slirp* heterozygous KO (*Slirp* ^+/-^) animals. The *Slirp* ^+/-^ mice were then backcrossed to C57BL/6N mice for seven generations and intercrosses were used to generate wild-type (WT, *Slirp* ^+/+^) and *Slirp* homozygous KO (*Slirp* ^-/-^) animals.

### Ethics statement

This study was performed in strict accordance with the recommendations and guidelines of the Federation of European Laboratory Animal Science Associations (FELASA). The protocol was approved by the “Landesamt für Natur, Umwelt und Verbraucherschutz Nordrhein-Westfalen”.

The mice were housed in specific pathogen-free conditions with a 12 hr light-dark cycle and had free access to water and food. Phenotypical characterization was performed at the German Mouse Clinic on 15 *Slirp* ^+/+^ and 15 *Slirp* ^-/-^ mice of each gender aged between 9 and 21 weeks. At the German Mouse Clinic, the mice were maintained according to the GMC housing conditions and German laws and the tests were performed as outlined in the standard operating procedures (SOP) linked to the EMPReSS website http://empress.har.mrc.ac.uk

### Mitochondrial transcript poly(A) tail length measurement (MPAT assay)

The MPAT assay was adapted from previous protocols [15,35]. RNA was extracted with TRIzol Reagent (Invitrogen) from heart mitochondria. An adaptor DNA oligonucleotide (sequence in S1 Table) was phosphorylated by the T4 PNK (New England Biolabs) and 2.5 pmol of this phosphorylated adaptor DNA oligonucleotide was then ligated to the 3’ end of 0.3 μg of total mitochondrial RNA, for each RNA species to be tested. The ligation reaction was performed using the T4 RNA ligase (New England Biolabs) for 2 hrs at 37°C. The ligated RNA was extracted with the TRIzol Reagent and reverse transcribed using the High capacity cDNA reverse transcription kit (Applied Biosystems) and a primer specific of the adaptor DNA oligonucleotide sequence (anti-adaptor, sequence in S1 Table). A first round of PCR was carried out for 29 cycles using a gene-specific upper primer and the anti-adaptor primer. The PCR products were purified on G-50 micro columns (GE Healthcare) in order to remove the primers. A nested PCR was then carried out for 12 cycles using a gene-specific lower primer and an inner anti-adaptor primer (sequence in S1 Table), in order to improve specificity. The PCR products were then cloned into the pCR4-TOPO vector and transformed into chemically-competent bacteria. Finally, DNA from selected colonies was extracted and sequenced in order to assess the length of the poly(A) stretch on the 3’ end of each mt-mRNA species.

### Transcription and translation assays

*In organello* transcription and translation assays were performed on mitochondria isolated from mouse tissues by differential centrifugation as detailed in the S1 Text. Mitochondria, 800 μg, were collected for each *in organello* transcription assay and washed in 1 ml of transcription buffer (10 mM Tris pH 7.4, 25 mM sucrose, 75 mM sorbitol, 100 mM KCl, 10 mM K_2_HPO_4_, 50 μM EDTA, 5 mM MgCl_2_, 10 mM glutamate, 2.5 mM malate, 1 mg/ml BSA and 1 mM ADP). An aliquot of mitochondria was collected for immunoblotting with the VDAC antibody (Millipore) to ensure equal loading. The remaining mitochondria were pelleted by centrifugation at 10,000 *g*, for 3 min at 4°C, suspended in 750 μl of transcription buffer supplemented with 30 μCi of ^32^P-UTP (PerkinElmer) and incubated for 20 min at 37°C. After the incubation, mitochondria were washed once and suspended in 750 μl of fresh transcription buffer in the presence of 0.2 mM of cold UTP. A short chase was performed for 5 min at 37°C in order to decrease the background and mitochondria were washed three times in 10 mM Tris pH 6.8, 0.15 mM MgCl_2_ and 10% glycerol. The mitochondrial pellet was suspended in 1 ml TRIzol (Invitrogen) for RNA extraction according to the manufacturer’s instructions. The isolated RNAs were analyzed by northern blotting and the radiolabeled transcripts were visualized by autoradiography.

Mitochondria, 500 μg, were collected for *in organello* translation assays and incubated in 750 μl translation buffer (100 mM mannitol, 10 mM sodium succinate, 80 mM KCl, 5 mM MgCl_2_, 1 mM KPi, 25 mM HEPES pH 7.4, 5 mM ATP, 20 μM GTP, 6 mM creatine phosphate, 60 μg/ml creatine kinase and 60 μg/ml of all amino acids except methionine). An aliquot of the mitochondrial preparation was set aside for immunoblotting to ensure equal loading as described above. Mitochondria were supplemented with 150 μCi of ^35^S methionine (PerkinElmer) for 10, 30 or 60 min at 37°C. After labeling, mitochondria were washed in translation buffer and suspended in a SDS-PAGE loading buffer. Translation products were resolved by SDS-PAGE and analyzed by autoradiography.

The mitochondrial translation rate was also assessed in cultured primary MEFs following a previously described method [36]. The translation products were labeled for 10, 30 and 60 min with 80 μCi/ml of a mixture of ^35^S-methionine/cysteine (Perkin Elmer) in DMEM lacking methionine and cysteine and in the presence of 100 μl/ml of the cytoplasmic translation inhibitor emetine (Sigma). After pulse labeling, a short chase was performed for 5 min at 37°C to decrease the background. The cells were then washed and lysed with RIPA lysis buffer. Protein concentration was measured and 50 μg of total cell extracts were resolved by SDS-PAGE and analyzed by autoradiography.

### Cell culture and RNAi

Knockdown of the mitochondrial LONP1 protease was performed on *Slirp* ^+/+^ *and Slirp* ^-/-^ primary MEFs plated on 10 cm diameter dishes. MEFs at 80% confluence were transfected with 1.4 μg of either scrambled or Lonp1 siRNA (Stealth siRNA negative control, Med. GC and Stealth siRNA Lonp1 respectively, Life technologies) in 12 μl of Lipofectamine RNAi Max (Invitrogen) per dish. Cells were harvested after 72 hrs either in TRIzol Reagent (Invitrogen) for RNA extraction or in RIPA lysis buffer [37] for total cell protein extraction.

### Northern blotting and qRT-PCR

For detection of mitochondrial RNAs, northern blotting and qRT-PCR were performed as described in the S1 Text.

### Immunoblotting

Protein steady-state levels were assessed by immunoblotting as described in the S1 Text.

### Sucrose density gradients

Heart and liver mitochondria, 1.2 mg, were lysed in the presence of 1% n-Dodecyl β-D-maltoside (Sigma). Lysates were loaded on 10–30% sucrose gradients and separated by centrifugation overnight as previously described [17,38]. Gradient fractions were collected as 750 μl aliquots. RNA was extracted from one third of each fraction by using the TRIzol LS Reagent (Invitrogen) according to the manufacturer’s recommendations. The samples were subsequently treated with DNase I and used for cDNA synthesis. The transcript abundance in each fraction was assessed by qRT-PCR analysis using the Taqman probes listed in S1 Table. The remaining two thirds of each fraction (500 μl) were precipitated with trichloracetic acid, resolved by SDS-PAGE and ribosome-containing fractions were detected by immunoblotting using antibodies specific for individual proteins from the 28S (MRPS35, Proteintech) and 39S (MRPL37, Sigma) ribosomal subunits.

### RNAseq

RNA from mitochondrial sucrose gradient fractions 6 and 9–12 was isolated and the concentration, purity, and integrity were confirmed using a BioAnalyser. The libraries were constructed using the Illumina TruSeq Sample Prep Kit and deep sequencing of the mitochondrial RNAs was performed by the Cologne Center for Genomics at the University of Cologne on an Illumina MiSeq according to the manufacturer’s instructions. Raw sequencing reads were aligned to the mouse mitochondrial genome (chrM; mm10) with Bowtie2 v2.2.4 (-p 20—very-sensitive) [39]. Gene-specific read counts were summarised with featureCounts [40] from the Subread package v1.3.5-p4 (-b-T 20-s 2) using the Ensembl 78 (GENCODE VM4) annotation, modified to merge Nd4L/Nd4 and Atp8/Atp6 annotations into bicistronic transcripts. Raw fragment counts were normalised as fragments per kilobase per million mapped reads (FPKM) and expression changes calculated as log2 fold changes of FPKM values. Heat maps were made with gplots v2.15.0 and transcripts were hierarchically clustered by complete linkage of their euclidean distances with the hclust and dist functions of the base stats package in R v3.1.2.

### Respiratory chain function and complex activity

The mitochondrial oxygen consumption flux and the respiratory chain complex activities were measured as described in the S1 Text and in previous works [41].

## Supporting Information

S1 Fig Whole body homozygous knockout of *Slirp*. (A) Subcellular localization of the endogenous SLIRP protein in 143B cells. (B) Targeting strategy for the disruption of the *Slirp* gene in mice. A set of primers (orange) was designed to detect the wild-type (WT, +/+) allele and another set of primers (blue) was designed to detect the knockout (KO,-/-) allele generated after Cre recombination. (C) PCR analysis of a tail biopsy from 3-week old mice. WT pups showed a lack of DNA amplification with the KO allele primer set (blue) whereas *Slirp* homozygous KO pups showed a lack of DNA amplification with the WT allele primer set (orange). In the *Slirp* heterozygous KO mice (+/-), both the WT and the KO allele primer pairs generated bands at 383 bp and ~700bp, respectively. (D) Body weight of WT (*Slirp* ^+/+^, white bars) and homozygous *Slirp* KO (*Slirp* ^-/-^, black bars) mice measured in females and males at 11 weeks of age, n = 15. Error bars represent SEM. * p value < 0.05. (E) LRPPRC-Flag co-immunoprecipitation from heart and liver mitochondria of mice expressing the recombinant LRPPRC-Flag on a *Slirp* WT (*Slirp* ^+/+^) or KO (*Slirp* ^-/-^) background, followed by immunoblots against LRPPRC and SLIRP.
(TIF)

S2 Fig SLIRP absence affects mitochondrial mRNA stability. (A) MtDNA quantification by qPCR in heart, liver and kidney samples from 12-week old wild-type (WT, *Slirp* ^+/+^) and *Slirp* homozygous knockout (KO, *Slirp* ^-/-^) mice. n = 5, error bars represent SEM. (B) Mitochondrial transcript steady-state levels assessed by northern blotting in hearts from 12-week old WT (+/+) and homozygous *Slirp* KO (-/-) mice, as well as in hearts from 12-week old *Lrpprc* control (p/p) and conditional KO (p/p, cre) mice. (C) Mitochondrial transcript steady-state levels assessed by qRT-PCR in liver samples from 12-week old *Slirp* ^+/+^ and *Slirp* ^-/-^ mice. n = 5, error bars represent SEM. * p value < 0.05. ** p value < 0.01. *** p value < 0.001. (D) Distribution of the length of the poly(A) tails expressed in % of the total number of clones, with oligo(A) tail ≤ 10nt and poly(A) >10nt, in heart mitochondria from *Slirp* ^+/+^, *Slirp* ^-/-^, *Lrpprc* heterozygous KO (*Lrpprc* ^+/-^) and *Lrpprc* conditional KO (*Lrpprc* p/p, cre) mice.
(TIF)

S3 Fig Loss of SLIRP affects the mitochondrial ribosome profile. (A) Steady-state levels of the mitochondrial ribosomal proteins MRPL37 and MRPS35 were assessed by immunoblotting of protein extracts from heart and liver mitochondria from 12-week old *Slirp* ^+/+^ and *Slirp* ^-/-^ mice. VDAC was used as a loading control. (B) Steady-state levels of the mitochondrial translation initiation factors mtIF2 and mtIF3 as assessed by immunoblotting on heart and liver mitochondria from 12-week old *Slirp* ^+/+^ and *Slirp* ^-/-^ mice. VDAC was used as loading control. (C) Sedimentation profiles of transcripts and ribosomal proteins in sucrose density gradients of extracts from heart mitochondria from 12-week old *Slirp* ^+/+^ and *Slirp* ^-/-^ mice. Individual mitochondrial transcripts were detected by qRT-PCR. The RNA abundance is expressed as a percentage of the levels in the control. The migration of the small mitochondrial ribosomal subunit (28S) the large mitochondrial ribosomal subunit (39S) and the assembled mitochondrial ribosome (55S) was determined by assessing the profiles of the 12S and 16S rRNAs as well as the migration of MRPL37 protein of the large ribosomal subunit. (D) Individual mRNA sedimentation profiles from the gradient described in (C). *Slirp* ^+/+^ profiles are depicted in purple and *Slirp* ^-/-^ profiles are depicted in orange. Individual mitochondrial mRNAs were detected by qRT-PCR and the mRNA distribution profile is shown after normalization to controls.
(TIF)

S4 Fig The absence of SLIRP does not affect respiratory chain activity. (A) Mitochondrial translation rate assessed by *in organello* ^35^S-methionine pulse labelling for 10, 30 and 60 minutes in isolated kidney mitochondria from 12-week old wild-type (*Slirp* ^+/+^) and *Slirp* homozygous knockout (*Slirp* ^-/-^) mice. (B) Mitochondria-encoded respiratory chain subunit stability assessed by a 60 minutes *in cellulo* ^35^S-methionine/cysteine pulse labelling followed by 7 and 22 hrs chase in *Slirp* ^+/+^ and *Slirp* ^-/-^primary MEFs. (C) Oxygen consumption rates of isolated heart mitochondria from 12-week old *Slirp* ^+/+^ and *Slirp* ^-/-^ mice. Isolated mitochondria were incubated with complex I or complex II substrates. Each set of substrates was successively combined with ADP (to assess the phosphorylating respiration), oligomycin (to assess the non-phosphorylating respiration) and CCCP (to assess uncoupled respiration). n = 3. Error bars represent the SEM. (D) The activity of the respiratory chain complexes I (CI), II (CII) and IV (CIV) in heart mitochondria from 12-week old *Slirp* ^+/+^ and *Slirp* ^-/-^ mice. The citrate synthase activity (CS) was used as a control. n = 3. Error bars represent the SEM.
(TIF)

S1 Table List of primers and probes used in the study. (PDF)

S1 Text Supporting information. This section contains the supporting methods and the appendix.
(DOCX)
