# Supplementary figures and images for: SLIRP Regulates the Rate of Mitochondrial Protein Synthesis and Protects LRPPRC from Degradation
Source: PLoS Genet. 2015 Aug 6;11(8):e1005423. doi: 10.1371/journal.pgen.1005423 (PMC4527767; doi:10.1371/journal.pgen.1005423)

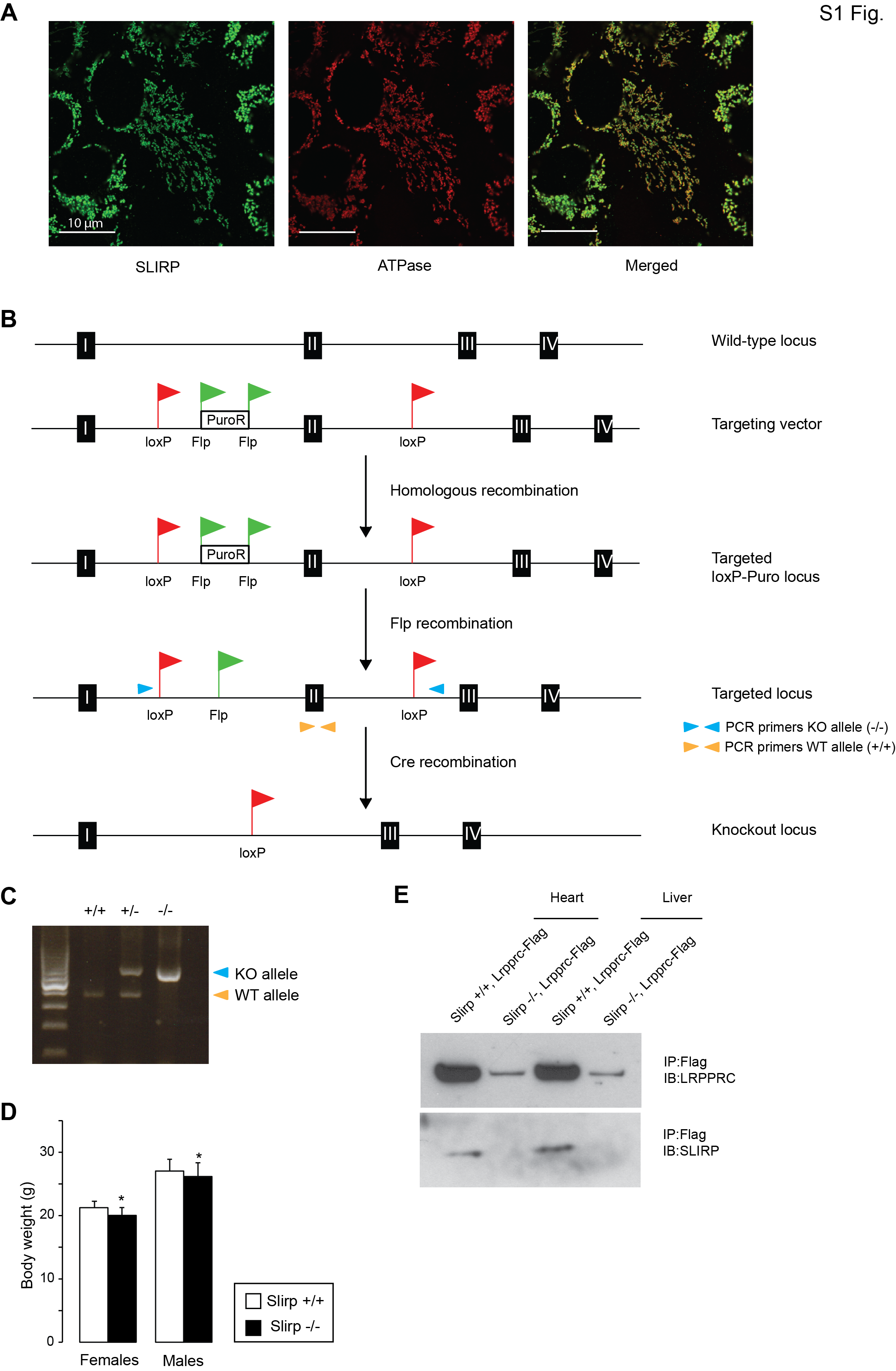

Supplement: S1 Fig — (A) Subcellular localization of the endogenous SLIRP protein in 143B cells. (B) Targeting strategy for the disruption of the Slirp gene in mice. A set of primers (orange) was designed to detect the wild-type (WT, +/+) allele and another set of primers (blue) was designed to detect the knockout (KO,-/-) allele generated after Cre recombination. (C) PCR analysis of a tail biopsy from 3-week old mice. WT pups showed a lack of DNA amplification with the KO allele primer set (blue) whereas Slirp homozygous KO pups showed a lack of DNA amplification with the WT allele primer set (orange). In the Slirp heterozygous KO mice (+/-), both the WT and the KO allele primer pairs generated bands at 383 bp and ~700bp, respectively. (D) Body weight of WT (Slirp +/+, white bars) and homozygous Slirp KO (Slirp -/-, black bars) mice measured in females and males at 11 weeks of age, n = 15. Error bars represent SEM. * p value < 0.05. (E) LRPPRC-Flag co-immunoprecipitation from heart and liver mitochondria of mice expressing the recombinant LRPPRC-Flag on a Slirp WT (Slirp +/+) or KO (Slirp -/-) background, followed by immunoblots against LRPPRC and SLIRP. (TIF) [file pgen.1005423.s001.tif]

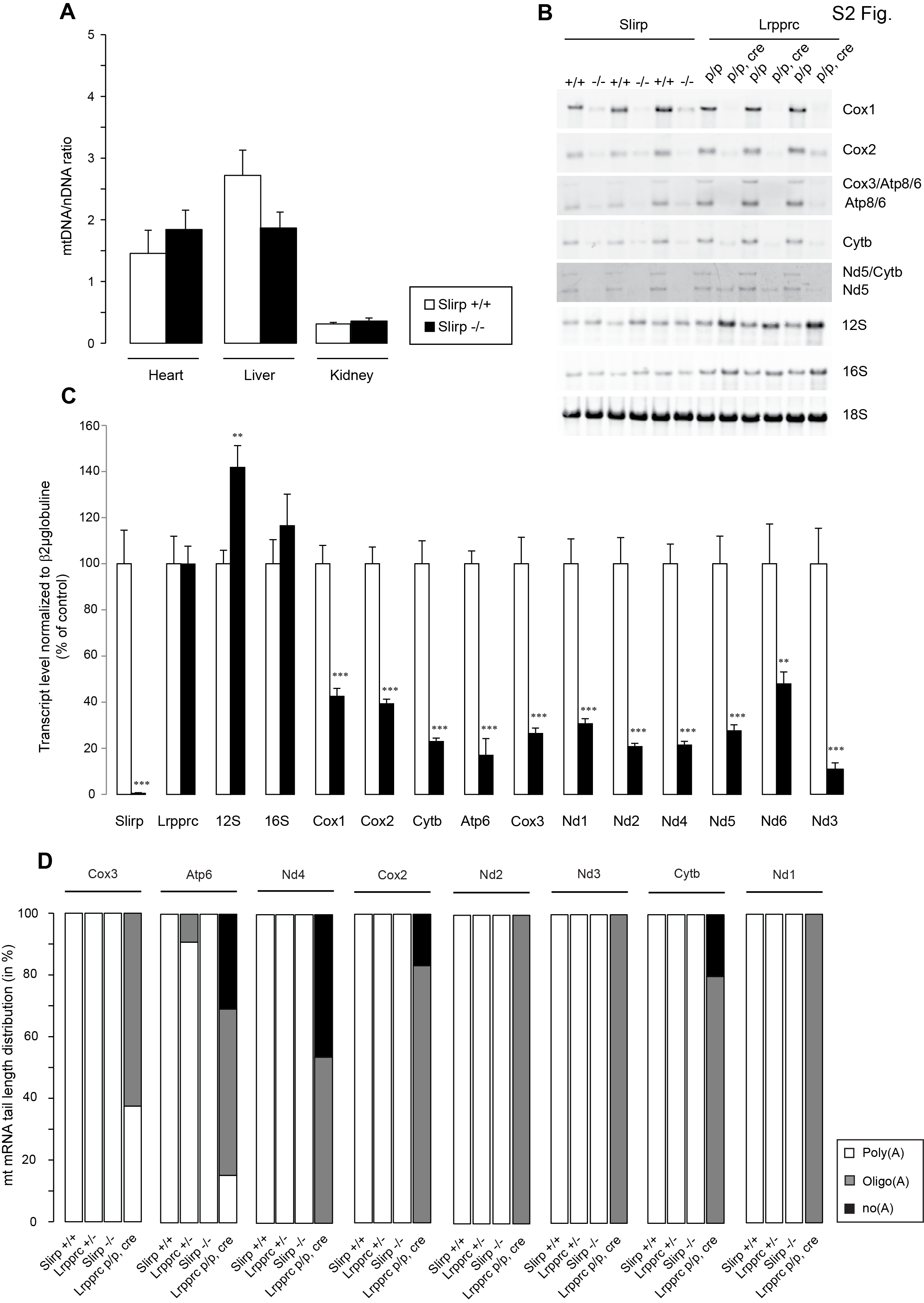

Supplement: S2 Fig — (A) MtDNA quantification by qPCR in heart, liver and kidney samples from 12-week old wild-type (WT, Slirp +/+) and Slirp homozygous knockout (KO, Slirp -/-) mice. n = 5, error bars represent SEM. (B) Mitochondrial transcript steady-state levels assessed by northern blotting in hearts from 12-week old WT (+/+) and homozygous Slirp KO (-/-) mice, as well as in hearts from 12-week old Lrpprc control (p/p) and conditional KO (p/p, cre) mice. (C) Mitochondrial transcript steady-state levels assessed by qRT-PCR in liver samples from 12-week old Slirp +/+ and Slirp -/- mice. n = 5, error bars represent SEM. * p value < 0.05. ** p value < 0.01. *** p value < 0.001. (D) Distribution of the length of the poly(A) tails expressed in % of the total number of clones, with oligo(A) tail ≤ 10nt and poly(A) >10nt, in heart mitochondria from Slirp +/+, Slirp -/-, Lrpprc heterozygous KO (Lrpprc +/-) and Lrpprc conditional KO (Lrpprc p/p, cre) mice. (TIF) [file pgen.1005423.s002.tif]

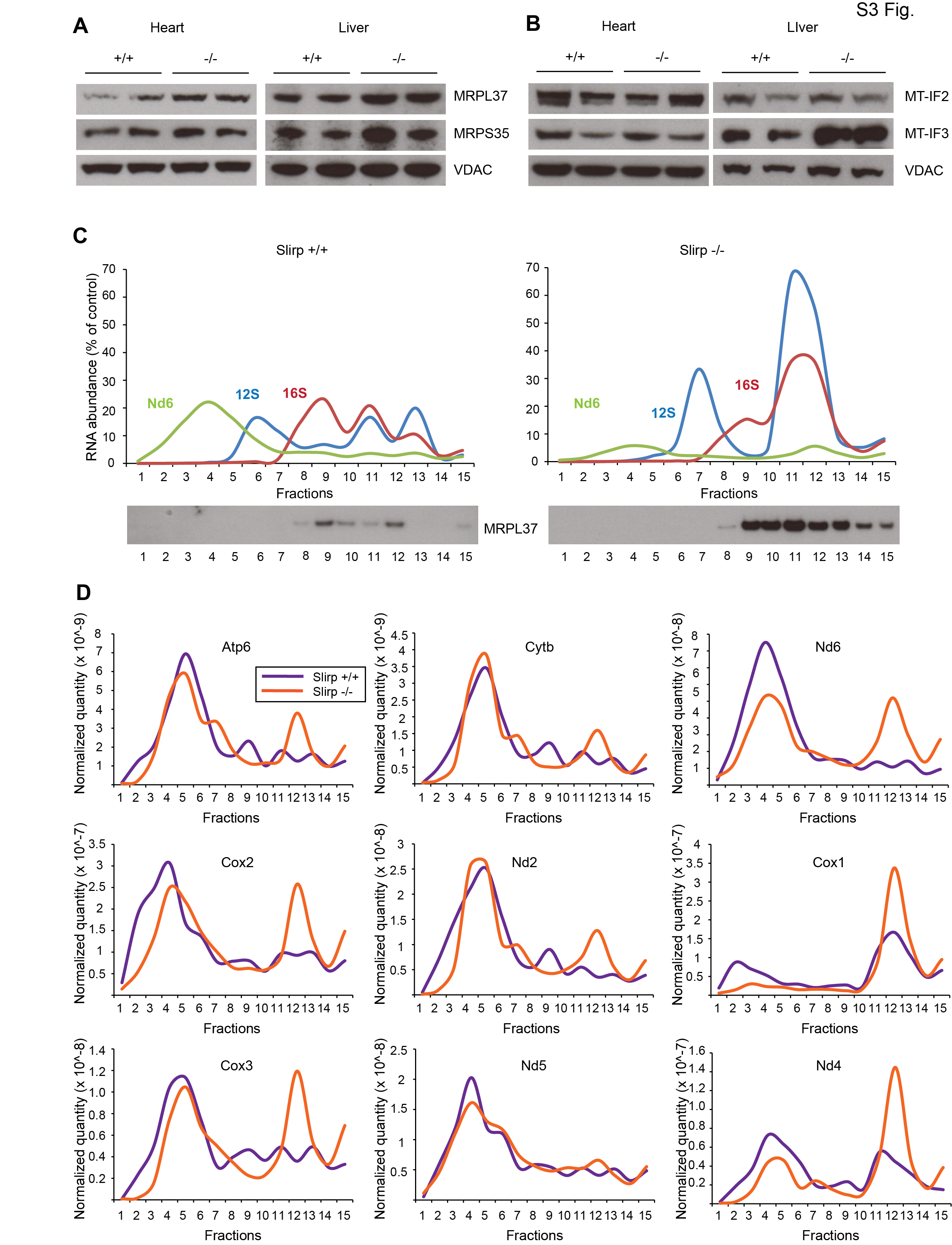

Supplement: S3 Fig — (A) Steady-state levels of the mitochondrial ribosomal proteins MRPL37 and MRPS35 were assessed by immunoblotting of protein extracts from heart and liver mitochondria from 12-week old Slirp +/+ and Slirp -/- mice. VDAC was used as a loading control. (B) Steady-state levels of the mitochondrial translation initiation factors mtIF2 and mtIF3 as assessed by immunoblotting on heart and liver mitochondria from 12-week old Slirp +/+ and Slirp -/- mice. VDAC was used as loading control. (C) Sedimentation profiles of transcripts and ribosomal proteins in sucrose density gradients of extracts from heart mitochondria from 12-week old Slirp +/+ and Slirp -/- mice. Individual mitochondrial transcripts were detected by qRT-PCR. The RNA abundance is expressed as a percentage of the levels in the control. The migration of the small mitochondrial ribosomal subunit (28S) the large mitochondrial ribosomal subunit (39S) and the assembled mitochondrial ribosome (55S) was determined by assessing the profiles of the 12S and 16S rRNAs as well as the migration of MRPL37 protein of the large ribosomal subunit. (D) Individual mRNA sedimentation profiles from the gradient described in (C). Slirp +/+ profiles are depicted in purple and Slirp -/- profiles are depicted in orange. Individual mitochondrial mRNAs were detected by qRT-PCR and the mRNA distribution profile is shown after normalization to controls. (TIF) [file pgen.1005423.s003.tif]

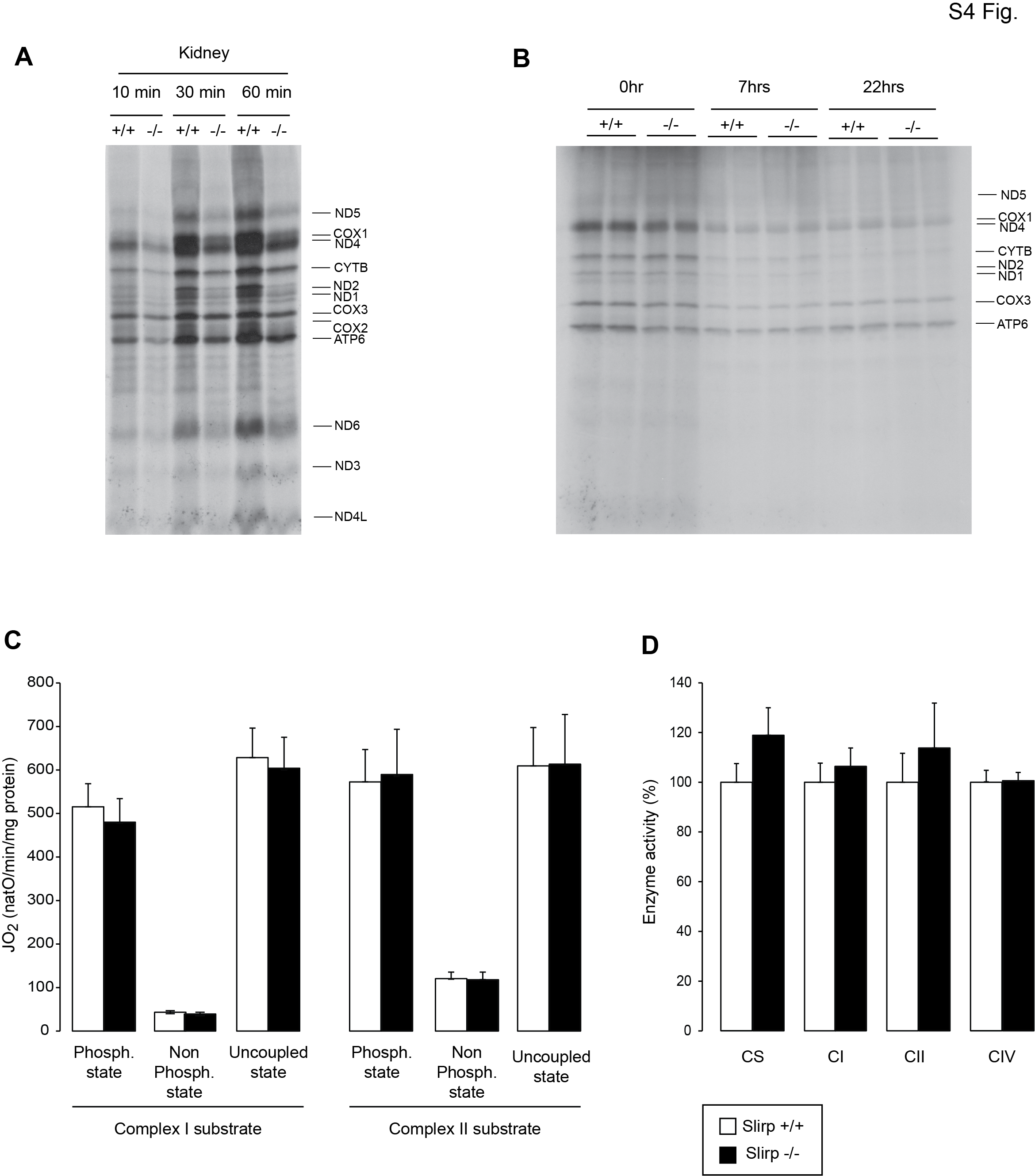

Supplement: S4 Fig — (A) Mitochondrial translation rate assessed by in organello 35S-methionine pulse labelling for 10, 30 and 60 minutes in isolated kidney mitochondria from 12-week old wild-type (Slirp +/+) and Slirp homozygous knockout (Slirp -/-) mice. (B) Mitochondria-encoded respiratory chain subunit stability assessed by a 60 minutes in cellulo 35S-methionine/cysteine pulse labelling followed by 7 and 22 hrs chase in Slirp +/+ and Slirp -/-primary MEFs. (C) Oxygen consumption rates of isolated heart mitochondria from 12-week old Slirp +/+ and Slirp -/- mice. Isolated mitochondria were incubated with complex I or complex II substrates. Each set of substrates was successively combined with ADP (to assess the phosphorylating respiration), oligomycin (to assess the non-phosphorylating respiration) and CCCP (to assess uncoupled respiration). n = 3. Error bars represent the SEM. (D) The activity of the respiratory chain complexes I (CI), II (CII) and IV (CIV) in heart mitochondria from 12-week old Slirp +/+ and Slirp -/- mice. The citrate synthase activity (CS) was used as a control. n = 3. Error bars represent the SEM. (TIF) [file pgen.1005423.s004.tif]
