# Supplementary material for: SLIRP Regulates the Rate of Mitochondrial Protein Synthesis and Protects LRPPRC from Degradation
Source: PLoS Genet. 2015 Aug 6;11(8):e1005423. doi: 10.1371/journal.pgen.1005423 (PMC4527767; doi:10.1371/journal.pgen.1005423)
Supplement: S1 Table — (PDF) [file pgen.1005423.s005.pdf]

S1 Table

**Genotyping primers**	
Slirp WT Fw	TGTATCTGGCAGGACACTGG
Slirp WT Rv	ATACTAGGCAAGCGCTCCAC
Slirp KO Fw	GAGGAGATGAGCGTACTGGC
Slirp KO Rv	CCC TCC CTGTGGGAGAGG
**Taqman probes for mtDNA quantification**	
16S	Mm03975671_s1
Atp6	Mm03649417_g1
18S	Hs99999901_s1
**Taqman probes for mRNA level measurement**	
b2µglobuline	Mm00437762_m1
Slirp	Mm01296845_m1
Lrpprc	Mm00511512_m1
12S	AJBJWSP
16S	Mm03975671_s1
Cox1	Mm04225243_g1
Cox2	Mm03294838_g1
Cox3	Mm04225261_g1
Cytb	Mm04225271_g1
Atp6	Mm03649417_g1
Nd1	Mm04225274_s1

Nd2	Mm04225288_s1
Nd4	Mm04225294_s1
Nd5	AIHSNT9
Nd6	AIVI3E8
**Oligonucleotides sequences for MPAT assay**	
Adaptor	ATGTGAGATCATGCACAGTCATA
Anti-adaptor	TATGACTGTGCATGATCTCACAT
Inner anti-adaptor	GACTGTGCATGATCTCACAT
Upper Cytb	ACGCCATTCTACGCTCAATC
Inner Cytb	CCTAATATTCCGCCCAATCA
Upper Nd1	TCCTATGGATCCGAGCATCT
Inner Nd1	GGATCCGAGCATCTTATCCA
Upper Nd2	GAGGCCTTCCACCACTAACA
Inner Nd2	GCCTTCCACCACTAACAGGA
Upper Nd3	TTCGACCCTACAAGCTCTGC
Inner Nd3	CAAGCTCTGCACGTCTACCA
Upper Nd4	CTATAATCATGGCCCGAGGA
Inner Nd4	AACCTCCAACCCTCACACAC
Upper Cox2	CAGTCCCCTCCCTAGGACTT
Inner Cox2	AGGCCGACTAAATCAAGCAA
Upper Cox3	TTACTTCACCATCCTCCAAGC
Inner Cox3	CACCATCCTCCAAGCTTCA

Upper Atp6	ACCAATGGCATTAGCAGTCC
Inner Atp6	TTAGCCCACCAACAGCTACC
