# Supplementary material for: SLIRP Regulates the Rate of Mitochondrial Protein Synthesis and Protects LRPPRC from Degradation
Source: PLoS Genet. 2015 Aug 6;11(8):e1005423. doi: 10.1371/journal.pgen.1005423 (PMC4527767; doi:10.1371/journal.pgen.1005423)
Supplement: S1 Text — This section contains the supporting methods and the appendix. (DOCX) [file pgen.1005423.s006.docx]

**SLIRP regulates the rate of mitochondrial protein synthesis**

**and protects LRPPRC from degradation**

Marie Lagouge, Arnaud Mourier, Hyun Ju Lee, Henrik Spåhr, Timothy Wai, Christian Kukat, Eduardo Silva Ramos, Elisa Motori, Jakob D. Busch, Stefan Siira, German Mouse Clinic Consortium, Elisabeth Kremmer, Aleksandra Filipovska^&^ and Nils-Göran Larsson^&^.

**Supporting Information**

**Supporting Methods**

**Bioinformatic prediction of SLIRP subcellular localization**

Subcellular localization of SLIRP was predicted with the MitoProt II 1.0a4 software (http://ihg.gsf.de/ihg/mitoprot.html).

**Immunocytochemistry**

For the detection of endogenous SLIRP, 143B cells were seeded on coverslips, fixed with 8% paraformaldehyde, incubated with an anti-SLIRP antibody (ab51523, Abcam) and visualized using a secondary Alexa Fluor 488 goat anti-rabbit antibody (Lifetech). The mitochondrial staining was achieved by anti-ATPase (Abcam) visualized by a secondary Alexa Fluor 594 goat anti-mouse antibody (Lifetech). The image acquisition was performed with a Leica TCS SP5-X inverted confocal microscope (Leica Microsystems) using a HCX PL APO CS 100x/1.46 oil objective.

For detection of endogenous LRPPRC, primary MEFs were fixed with 4% paraformaldehyde and incubated with the following primary antibodies: rabbit anti-LRPPRC (LRP-130, sc-66844 from Santa Cruz, 1:250) and mouse anti-TOM20 (WH0009804M1 from Sigma, 1:500). Visualization was performed by using the Alexa Fluor 594 goat anti-mouse antibody (Invitrogen) as a secondary antibody. The image acquisition was performed as described above.

**Mitochondria isolation from mouse tissues**

Mitochondria from heart, liver and kidney were isolated by differential centrifugation (2 x 10 min at 1,000 *g*, 4°C and 1 x 10 min at 10,000 *g*, 4°C) in mitochondrial isolation buffer (10 mM Tris pH 7.4, 320 mM Sucrose and 1 mM EDTA).

**mtDNA quantification**

DNA was extracted using the ‘DNeasy Tissue and Blood’ kit (Qiagen) according to the manufacturer’s instructions. Genotyping PCRs were performed using the primers listed in S1 Table. 5ng of total DNA from heart, liver and kidney were used for detection of mtDNA and genomic DNA by qPCR with the Taqman probes listed in S1 Table.

**Northern blotting and qRT-PCR**

RNA for northern blotting and qRT-PCR was isolated with TRIzol (Invitrogen) and suspended in nuclease-free water (Ambion). For detection of the mitochondrial RNAs by northern blotting, 2 µg of heart total RNA was denatured in NorthernMax-Gly Sample Loading Dye (Ambion) for 30 min at 50°C, separated on a agarose gel containing formaldehyde and transferred to Hybond-N+ nylon membrane (GE Healthcare). Oligonucleotides for the detection of the mt-tRNAs were end-labeled with 40 µCi of ^32^P-ATP by the T4 Polynucleotide Kinase (New England Biolabs). Probes for the detection of mt-mRNAs and mt-rRNAs were radiolabeled with 50 µCi of α-^32^P-dCTP using the Prime-It II random primer labeling kit (Stratagene). The radiolabeled probes were then incubated for 1 hr at 42°C (tRNAs) or at 65°C (mRNAs and rRNAs) with the nylon membrane and the radioactive signal was detected by autoradiography.

For detection of the mt-mRNAs and nuclear-encoded mitochondrial-related mRNAs by qRT-PCR, total RNA was treated with DNAse I using the TURBO DNA-free kit (Ambion) according to the manufacturer’s instructions. DNAse-treated RNAs, 2 µg, were reverse transcribed using the high capacity cDNA reverse transcription kit (Applied Biosystems) and qRT-PCR was performed on cDNAs using the Taqman Universal PCR Master Mix, No AmpErase UNG (Applied Biosystems) and the Taqman probes listed in S1 Table.

**Immunoblotting**

Immunoblotting was performed by using 50 µg of mitochondrial proteins isolated from mouse heart, liver and kidney or from total cell extracts that were resolved by SDS-PAGE and transferred to a nitrocellulose membrane. LRPPRC was detected using a polyclonal antiserum directed against human LRPPRC. The monoclonal antibody (15C4; rat IgG2a) directed against mouse SLIRP was generated using N-6xHis-SLIRP recombinant protein produced in *E.coli* using standard procedures. MRPL37 and MRPS35 antibodies were purchased from Sigma and Proteintech respectively. VDAC (Millipore) was used as a loading control. The anti-LONP1 antibody was a gift from the laboratory of Thomas Langer. SUV3L1 and PNPT1 antibodies were purchased from Abcam and Proteintech respectively and an antibody against SDHA (Invitrogen) was used as a loading control.

**Respiratory chain function and complex activity**

## The mitochondrial oxygen consumption flux was measured with an Oxygraph-2k (Oroboros Instruments) as previously described ([Mourier et al., 2014](#_ENREF_18)) at 37°C by using 65 to 125 μg of crude mitochondria diluted in 2.1 ml of mitochondrial respiration buffer (120 mM sucrose, 50 mM KCl, 20 mM Tris-HCl, 4 mM KH_2_PO_4_, 2 mM MgCl_2_, 1 mM EGTA, pH 7.2). The oxygen consumption rate was measured using either 10 mM pyruvate, 5 mM glutamate and 5 mM malate, or 10 mM succinate and 10 nM rotenone. Oxygen consumption was assessed in the phosphorylating state with 1 mM ADP or non-phosphorylating state by adding 2.5 μg/ml oligomycin. In the control mitochondria, the respiratory control ratio (RCR) values were >10 with pyruvate/glutamate/malate and >5 with succinate/rotenone. Respiration was uncoupled by successive addition of carbonyl cyanide m-chlorophenyl hydrazone (CCCP) up to 3 μM to reach maximal respiration.

## Mitochondria, 15–50 μg, were diluted in phosphate buffer (50 mM KH_2_PO_4_, pH 7.4) and spectrophotometric analyses of isolated respiratory chain complex activities were performed at 37 °C by using a Hitachi UV-3600 spectrophotometer. The citrate synthase activity was measured at 412 nm (*E* = 13,600 M^−1^ cm^−1^) after the addition of 0.1 mM acetyl-CoA, 0.5 mM oxaloacetate and 0.1 mM 5,5′-dithiobis-2-nitrobenzoic acid (DTNB). The SDH activity was measured at 600 nm (*E* = 21,000 M^−1^ cm^−1^) after the addition of 10 mM succinate, 35 μM dichlorophenolindophenol (DCPIP) and 1 mM KCN. The NADH dehydrogenase activity was determined at 340 nm (*E* = 6, 220 M^−1^ cm^−1^) after addition of 0.25 mM NADH, 0.25 mM decylubiquinone and 1 mM KCN, controlling for rotenone sensitivity. The COX activity was measured by standard N,N,N′,N′-tetramethylphenylene-1,4-diamine (TMPD) ascorbate assays, controlling for KCN sensitivity. All chemicals were obtained from Sigma-Aldrich.

**Statistical analyses**

The following statistical tests were performed using GraphPad Prism software:

Fig. 1D: Unpaired t-test to compare the *Slirp*^+/+^ *versus* *Slirp*^-/-^ conditions and *Lrpprc* p/p *versus* *Lrpprc* p/p, Cre conditions. Fig. 1E: Unpaired t-test with Welch’s correction to compare the *Slirp*^+/+^ and *Slirp*^-/-^ conditions. Figs. 3D and 3E: Unpaired t-test to compare the *Slirp*^+/+^ *versus* *Slirp*^-/-^ conditions. Fig. 4B: Unpaired t-test to compare the *Slirp*^+/+^ *versus* *Slirp*^-/-^ conditions and to compare the siCtrl and siLonp1 conditions in *Slirp*^-/-^ MEFs. Fig. 4C: One way ANOVA.

**Appendix**

^§^ German Mouse Clinic, Helmholtz Zentrum München, German Research Center for Environmental Health GmbH, Neuherberg, Germany

Thure Adler ^1,2^, Dirk H. Busch ^2^, Antonio Aguilar-Pimentel^1,3^, Markus Ollert^3, 11^, Oana Amarie^1,4^, Tobias Stoeger^1,4^, Ali Önder Yildrim^1,4^, Oliver Eickelberg^4^, Lore Becker^1,5^, Alexandra Vernaleken^1, 5^, Thomas Klopstock ^5,16,20,21^, Marion Horsch^1^, Johannes Beckers^1,18,19^, Kristin Moreth^1^, Raffi Bekeredjian^6^, Hugo Katus^6^, Lillian Garrett^1,8^, Sabine M. Hölter ^1,8^, Annemarie Zimprich^1,8^, Wolfgang Wurst ^8,14,15,16,17,21^, Oliver Puk^1,8^, Jochen Graw ^8^, Wolfgang Hans^1^, Jan Rozman ^1,19^, Martin Klingenspor^9,10^, Laura Brachthäuser^1,7^, Julia Calzada-Wack^1,7^, Dirk Janik^1,7^, Tanja Klein-Rodewald^1,7^, Frauke Neff^1,7^, Ildikó Rácz^1,12^, Andreas Zimmer^12^, Birgit Rathkolb ^1,13,19^, Eckhard Wolf ^13^, Manuela Östereicher^1^, Ralph Steinkamp^1^, Christoph Lengger^1^, Holger Maier^1^, Claudia Stoeger^1^, Stefanie Leuchtenberger^1^, Valérie Gailus-Durner ^1^, Helmut Fuchs ^1^, Martin Hrabě de Angelis ^1,18,19^

^1^ German Mouse Clinic, Institute of Experimental Genetics, Helmholtz Zentrum München, German Research Center for Environmental Health GmbH, Ingolstaedter Landstrasse 1, 85764 Neuherberg, Germany

^2^ Institute for Medical Microbiology, Immunology and Hygiene, Technical University of Munich, Trogerstrasse 9, 81675 Munich, Germany

^3^ Department of Dermatology and Allergy, Biederstein, Klinikum rechts der Isar, Technische Universität München (TUM), Biedersteiner Str. 29, 80802 Munich,

^4^ Comprehensive Pneumology Center, Institute of Lung Biology and Disease, Helmholtz Zentrum München, German Research Center for Environmental Health (GmbH), Ingolstädter Landstraße 1, 85764 Neuherberg, Germany and Member of the German Center for Lung Research

^5^ Department of Neurology, Friedrich-Baur-Institut, Ludwig-Maximilians-Universität München, Ziemssenstrasse 1a, 80336 Munich, Germany

^6^ Department of Cardiology, University of Heidelberg, Im Neuenheimer Feld 410, 69120 Heidelberg, Germany

^7^ Institute of Pathology, Helmholtz Zentrum München, German Research Center for Environmental Health GmbH, Ingolstaedter Landstrasse 1, 85764 Neuherberg, Germany

^8^ Institute of Developmental Genetics, Helmholtz Zentrum München, German Research Center for Environmental Health GmbH, Ingolstaedter Landstrasse 1, 85764 Neuherberg, Germany

^9^ Chair for Molecular Nutritional Medicine, Technische Universität München, Else Kröner-Fresenius Center for Nutritional Medicine, 85350 Freising, Germany

^10^ ZIEL – Center for Nutrition and Food Sciences, Technische Universität München, 85350 Freising, Germany

^11^ Clinical Research Group Molecular Allergology, Center of Allergy and Environment Munich (ZAUM), Technische Universität München (TUM), and Institute for Allergy Research, Helmholtz Zentrum München, German Research Center for Environmental Health, Neuherberg, Germany

^12^ Institute of Molecular Psychiatry, University of Bonn, Sigmund-Freud-Strasse 25, 53127 Bonn, Germany

^13^ Ludwig-Maximilians-Universität München, Gene Center, Institute of Molecular Animal Breeding and Biotechnology, Feodor-Lynen Strasse 25, 81377 Munich, Germany

^14^ Chair of Developmental Genetics, Center of Life and Food Sciences Weihenstephan, Technische Universität München, Ingolstaedter Landstrasse 1, 85764 Neuherberg, Germany

^15^ Max Planck Institute of Psychiatry, Kraepelinstr. 2-10 , 80804 Munich, Germany

^16^ Deutsches Institut für Neurodegenerative Erkrankungen (DZNE) Site Munich, Schillerstrasse 44, 80336 Munich, Germany

^17^ Munich Cluster for Systems Neurology (SyNergy), Adolf-Butenandt-Institut, Ludwig-Maximilians-Universität München, Schillerstrasse 44, 80336 Munich, Germany

^18^ Chair of Experimental Genetics, Center of Life and Food Sciences Weihenstephan, Technische Universität München, Ingolstaedter Landstrasse 1, 85764 Neuherberg, Germany

^19^ Member of German Center for Diabetes Research (DZD), Ingolstaedter Landstraße 1, 85764 Neuherberg, Germany

^20^ German Network for Mitochondrial Disorders (mitoNET)

^21^ German Center for Vertigo and Balance Disorders, Munich, Germany
